# Eosinophilic granulomatous polyangiitis with IgG4 hypergammaglobulinaemia and salivary gland swelling

**DOI:** 10.1002/rcr2.552

**Published:** 2020-03-30

**Authors:** Koichiro Takahashi, Hironori Sadamatsu, Hiroki Tashiro, Go Kato, Masaru Uchida, Naoko Sueoka‐Aragane

**Affiliations:** ^1^ Division of Haematology, Respiratory Medicine and Oncology, Department of Internal Medicine, Faculty of Medicine Saga University Saga Japan

**Keywords:** Eosinophilic granulomatous polyangiitis, IgG4, salivary gland

## Abstract

A 51‐year‐old woman was admitted to our hospital for cough, fever, purpura in the legs, and salivary gland swelling. Six years ago, she had been diagnosed with bronchial asthma and was treated with a combination of inhaled corticosteroid and long‐acting beta2‐agonist. Blood examination showed increased eosinophils at 3027 cells/μL and elevated levels of immunoglobulin (Ig) G4 at 261 mg/dL and C‐reactive protein at 2.76 mg/dL. Chest radiograph and computed tomography (CT) showed infiltrates in the bilateral lower lobes. Neck CT showed bilateral salivary gland swelling. Pathological examinations of the lungs and skin purpura showed granuloma with eosinophilic infiltration and perivascular dermatitis, respectively. She was diagnosed with eosinophilic granulomatous polyangiitis (EGPA) and treated with corticosteroids, which resolved the eosinophilia, salivary gland swelling, elevated IgG4 titre, and lung infiltration. As our patient did not meet the American College of Rheumatology/European League Against Rheumatism (ACR/EULAR) 2019 criteria of IgG4‐related disease, the diagnostic was EGPA with IgG4 hypergammaglobulinaemia and salivary gland swelling.

## Graphical abstract

We present a case of a 51‐year‐old Japanese woman diagnosed with eosinophilic granulomatous polyangiitis (EGPA), a rare vasculitis that can occur in patients with eosinophilia and history of asthma, allergic rhinitis, and sinusitis, with immunoglobulin (Ig) G4 hypergammaglobulinaemia and salivary gland swelling.

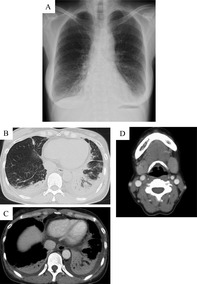

## Introduction

Eosinophilic granulomatous polyangiitis (EGPA) is a rare vasculitis that can occur in patients with eosinophilia and history of asthma, allergic rhinitis, and sinusitis. Immunoglobulin (Ig) G4‐related disease is a systemic immune‐mediated fibroinflammatory condition that involves multiple organs and is characterized by markedly increased serum IgG4 level, lymphoplasmacytic infiltration with abundant IgG4‐positive plasma cell, storiform fibrosis, and obliterative phlebitis. The diagnostic for the present case was EGPA with IgG4 hypergammaglobulinaemia and salivary gland swelling.

## Case Report

A 51‐year‐old Japanese woman was admitted to our hospital because of cough and fever for one month. She had been diagnosed with bronchial asthma and was treated with a combination of inhaled corticosteroid and long‐acting beta2‐agonist. Her asthma had been partly controlled. Although she had had unplanned visits for asthma exacerbation once or twice a year, she had no prior requirements for systemic corticosteroids. She was a never smoker and had no dust exposure. On admission, her body temperature was 38.1°C, and wheezes were heard in the bilateral lung fields. There were bilateral salivary gland swelling, leg purpura that measured 2–5 mm in diameter, and swelling of the left ankle joint. The other physical examination findings, including consciousness, heart sound, and abdomen, were normal. She had no reduction in the production of saliva or lacrimal fluid.

**Figure 1:**
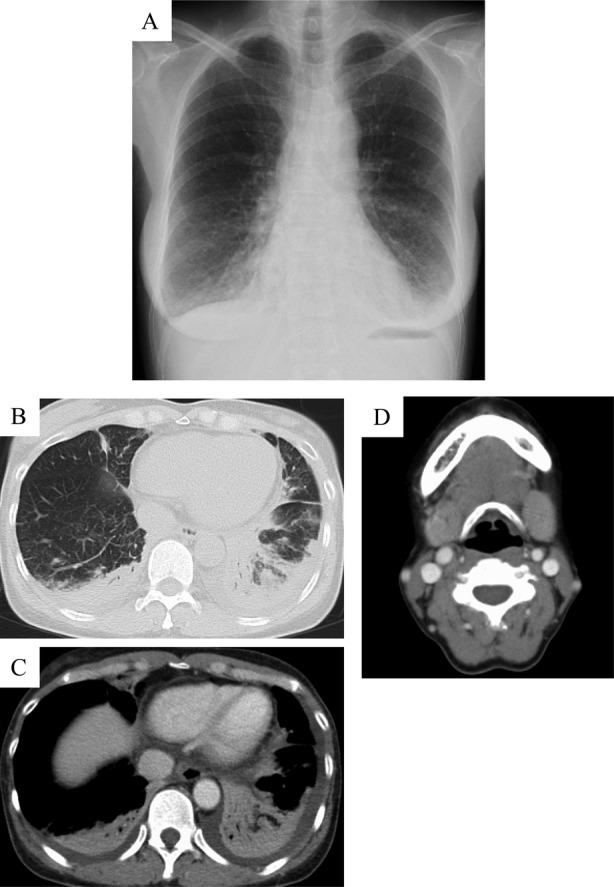
Chest radiography and computed tomography (CT) findings in a 51‐year‐old woman with eosinophilic granulomatous polyangiitis (EGPA). (A) Chest radiography shows infiltrates in the bilateral lower lung fields. (B, C) Chest CT shows dense infiltrations in the bilateral lower lobe and bilateral pleural effusion. (D) Head CT shows marked swelling of the bilateral submandibular glands.

**Figure 2:**
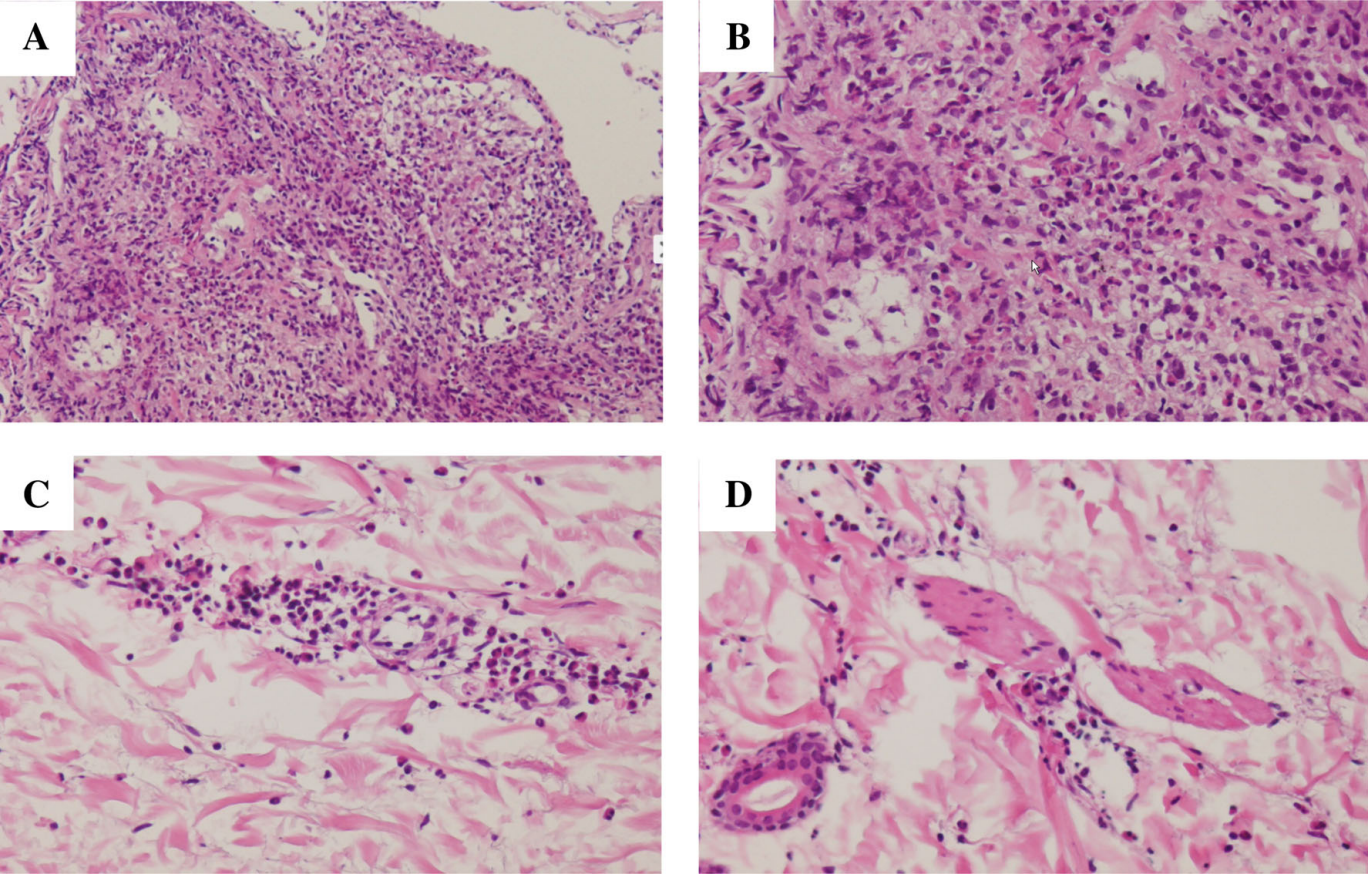
Pathological findings in a 51‐year‐old woman with eosinophilic granulomatous polyangiitis. (A, B) In the lungs, there are inflammatory granulations with marked eosinophil infiltration (haematoxylin–eosin stain: A, 40×; B, 100×). (C, D) In the skin, there is eosinophilic infiltration and perivascular dermatitis (haematoxylin–eosin stain: C, 100×; D, 100×).

Her laboratory findings showed white blood cell count of 9200/μL with 32.9% eosinophils, haemoglobin of 13.4 g/dL, C‐reactive protein of 2.76 mg/dL, IgE of 3052 IU/mL, IgG of 1235 mg/dL, IgG4 of 261 mg/dL, and rheumatoid factor of 1818 IU/mL. The other blood examinations, including myeloperoxidase antineutrophil cytoplasmic antibody (ANCA), proteinase‐3 (PR3)‐ANCA, anti‐SSA/Ro antibody, and anti‐SSB/La antibody, were within the normal range. Arterial blood gas at room air examination showed pH of 7.42, partial pressure of carbon dioxide (PaCO_2_) of 37.1 mmHg, partial pressure of oxygen (PaO_2_) of 68.5 mmHg, and bicarbonate (HCO_3_^−^) of 24.2 mEq/L. Chest radiograph showed infiltrations in the bilateral lower lung fields (Fig. 1A). Chest computed tomography (CT) showed infiltrations in the bilateral lower lobes and pleural effusion (Fig 1B, C). Neck CT showed marked bilateral submandibular gland swelling (Fig. 1D). She had no abdominal findings including pancreas, bile duct, kidney, and retroperitoneum lesion suggestive of IgG4‐related disease. Pathological findings of the transbronchial biopsy specimen showed inflammatory granulations with eosinophil infiltration (Fig. 2A, B), and those of the skin biopsy showed granuloma with eosinophilic infiltration and perivascular dermatitis (Fig. 2C, D). There was no plasma cell infiltration or IgG4‐positive cells in the lungs and skin. Biopsy of the salivary glands was not performed due to technical difficulties.

On the basis of the findings, she was diagnosed with EGPA and was initially treated with oral corticosteroids (prednisolone 1.0 mg/kg/day). Thereafter, there were resolution of fever, wheezes, and lung infiltrations on chest radiography and improvement of the IgG4 titre and salivary gland swelling. Concurrently, she was treated with intravenous cyclophosphamide every three weeks for three months. Thereafter, prednisolone was gradually reduced over a period of six months and had been maintained at 10 mg/day for three years. She had been relapse‐free at the time of this writing.

## Discussion

EGPA is a vasculitis that can occur in patients with eosinophilia and history of asthma, allergic rhinitis, and sinusitis. The histological features of EGPA include eosinophil‐rich infiltrates, eosinophilic granulomas, and vasculitis of small‐ to medium‐sized vessels. Based on the predisposition of patients with allergic diseases, the pathophysiological conditions of EGPA have been related to T‐helper type 2 (Th2)‐induced inflammation. Moreover, in patients with EGPA, the levels of Th2 cytokines, interleukin‐4 (IL‐4), IL‐5, and IL‐13 are elevated and approximately 90% have elevated serum IgE levels. On the other hand, IgG4‐related diseases, including sclerosing pancreatitis, retroperitoneal fibrosis, and chronic sialadenitis, have been characterized by IgG4‐positive plasma cell infiltration and high serum IgG4 levels 1. In this report, we presented a case of EGPA with high serum IgG4 and salivary gland swelling. According to ACR/EULAR 2019 criteria of IgG4‐related disease, the present case did not meet IgG4‐related disease 2. Therefore, the diagnostic for the present case was EGPA complicated with reactive IgG4 hypergammaglobulinaemia and salivary gland swelling.

Several cases of overlapping IgG4‐related disease and EGPA have been reported. Ayuzawa et al. reported a similar case of EGPA complicated with IgG4‐related kidney disease and who had salivary gland swelling and high serum IgG4 level at the time of EGPA diagnosis 3. Zhou et al. reported a case of EGPA complicated with abundant pulmonary IgG4‐positive plasma cell infiltration and markedly elevated serum IgG4 levels, without the extrapulmonary lesions of IgG4‐related diseases 4. Therefore, EGPA and IgG4‐related diseases have been reported to overlap at various rates. Danlos et al. reported 18 patients who had overlapping manifestations of ANCA‐associated vasculitides (AAV) and IgG4‐related disease 5. In that study, the AAV included granulomatosis with polyangiitis in 14 (78%), microscopic polyangiitis in three (17%), and eosinophilic granulomatosis with polyangiitis in one case (5%), suggesting that AAV and IgG4 may overlap.

In addition, a previous study that investigated the serum levels of IgG, IgM, IgA, IgE, and IgG subclasses in patients with active EGPA, quiescent EGPA, granulomatous with polyangiitis, atopic asthma, and healthy controls reported that the serum IgG4 levels were elevated in active EGPA and correlated with the number of organ manifestations and disease activity 6. That study suggested that serum IgG4 could be a marker of EGPA disease activity.

In conclusion, because EGPA could be complicated with reactive IgG4 hypergammaglobulinaemia and salivary gland swelling, measurements of serum IgG4 level and assessment of salivary glands should be performed in patients with EGPA.

### Disclosure Statement

Appropriate written informed consent was obtained for publication of this case report and its accompanying images.
